# Removal of Polystyrene Microplastics from Aqueous Solution Using the Metal–Organic Framework Material of ZIF-67

**DOI:** 10.3390/toxics10020070

**Published:** 2022-02-04

**Authors:** Hongyou Wan, Junkai Wang, Xiaoyu Sheng, Jingwei Yan, Wei Zhang, Ying Xu

**Affiliations:** 1 School of Ecology and Environment, Zhengzhou University, 100 Kexue Avenue, Zhengzhou 450001, China; hywan@zzu.edu.cn (H.W.); 13014502079@163.com (J.W.); sxy15670527525@163.com (X.S.); yanjingweide@163.com (J.Y.); 2 Research Centre of Engineering and Technology for Synergetic Control of Environmental Pollution and Carbon Emissions of Henan Province, Zhengzhou 450001, China; 3 Henan International Joint Laboratory of Water Cycle Simulation and Environmental Protection, Zhengzhou 450001, China; 4 Zhengzhou Key Laboratory of Water Resource and Environment, Zhengzhou 450001, China; 5 Yellow River Institute for Ecological Protection and Regional Coordination Development, Zhengzhou University, Zhengzhou 450001, China; 6 Henan Key Laboratory of Water Pollution Control and Rehabilitation Technology, Pingdingshan 467036, China; 7 Henan Key Laboratory of Water Resources Conservation and Intensive Utilization in the Yellow River Basin, Zhengzhou 450001, China

**Keywords:** microplastics, MOF material, ZIF-67, adsorption, water environment

## Abstract

Due to the continuous and adverse effects of microplastics on the environment, an increasing number of studies have begun to focus on their migration patterns and removal from aquatic environments. Herein, our study innovatively evaluated the ability of the capacity of ZIF-67, a novel metal–organic framework (MOF) material, to adsorb polystyrene (PS) microplastics (MPs) from aqueous solutions, aiming to explore the potential of MOF materials to remove MPs from wastewater. The adsorption ratio of PSMPs (5 mg/L, 30 mL) by ZIF-67 reached up to 92.1%, and the PSMP adsorption equilibrium was achieved within 20 min at 298 K. The adsorption of PSMPs would be favored at a pH of 8, a PSMPs solution concentration of 5 mg/L, and a temperature of 298 K. Further analyses demonstrated that hydrogen bond interactions, π-π stacking, and electrostatic interactions played a crucial role in the adsorption of PSMPs by ZIF-67 in aqueous solutions. Our findings thus provide insight into novel methods to remove MPs from acidic and weakly alkaline aquatic environments and wastewater.

## 1. Introduction

Worldwide plastic production reached 368 million tons in 2019, and these outputs are projected to reach 3.3 billion tons by 2050 [1]. These increases in plastic production and consumption will inevitably lead to increases in the discharge of plastic waste. In natural water or soil environments, microplastics (MPs, with size ranging from 1 μm to 5 mm) are generated from the degradation of plastic products through natural processes such as UV photodegradation, mechanical degradation, and hydrolysis [2,3,4,5]. MPs have become extremely conspicuous and have already been detected in personal care products, food (e.g., drinking water, milk, and table salt), and baby products [6,7,8,9]. Additionally, MPs are known to adsorb many types of pollutants such as heavy metals, persistent organic pollutants (POPs), and chemical additives such as flame retardants and antioxidants [2,10,11,12,13,14,15,16,17], thus exacerbating their adverse effects on human and animal health [18,19,20,21,22]. For example, after ingesting microplastics, the marine invertebrates’ digestive enzyme systems would be damaged, and even their reproductive system might be affected [19,23]. Furthermore, microplastics can accumulate in the human body and lead to obesity, cancer, and infertility [8,24,25,26]. As reported, microplastics have already been detected in wastewater in large amounts (0.05 ± 0.024 n/L), including plastic particles, fibers, fragments, films, and granules [27,28]. Microplastics will inevitably discharge into the aquatic environment, and further affect the survival of animals, plants, and microorganisms [29,30]. Several methods have been developed to remove MPs from wastewater, including filtration, coagulation, foam flotation, and magnetic separation [31,32,33,34,35]. However, these methods cannot efficiently remove smaller particle size plastics (<10 μm). Other studies have employed chitin sponge or biochar to adsorb MPs from aquatic environments [36,37]. Nevertheless, additional research is still required to identify novel methods and mechanisms to remove MPs (<10 μm) from water environments.

Metal–organic framework (MOF) materials have recently garnered increasing attention due to their applicability in water remediation [38]. Among these materials, zeolitic imidazolate frameworks (ZIF) are particularly promising, owing to their many advantages [11,39]. Specifically, these materials can be easily prepared, and possess a large specific surface area, stable structure, and large adsorption capacity [40,41,42]. ZIF-67 is a porous material, with CO^2+^ and 2-methylimidazole acting as the central metal ion and organic ligand, respectively [43,44]. Previous research has demonstrated that ZIF-67 could effectively adsorb and remove pollutants (e.g., organic pollutants, heavy metals, and antibiotics) from water through interface forces, including hydrogen bonding, electrostatic action, and π-π stacking [39,45,46,47]. However, very few studies have focused on the capacity of ZIF-67 to remove PSMPs from aqueous solutions.

Our study thus evaluated the PSMPs (<10 μm) removal performance of ZIF-67 in aqueous solutions. Specifically, this study assessed the key effects of ZIF-67 dose, contact time, pH, contact temperature, and PSMPs concentration on the adsorption process to explore the PSMP removal capacity of ZIF-67 in aqueous solution, as well as its potential adsorption mechanisms. We hypothesized that ZIF-67 could effectively adsorb and remove MPs from water through hydrogen bonding, electrostatic action, and π-π stacking, which could serve as the basis for novel strategies to treat MP contaminated wastewater.

## 2. Materials and Methods

### 2.1. Chemicals and Materials

Fluorescent PSMPs (with average diameter of 1.0 μm, 1% *w*/*v*) were purchased from the Tianjin Beisiline Chromtech Research Center, (Tianjin, China). The fluorescent PSMPs stock solution was diluted using deionized water, then treated with an ultrasonic device (KQ-100DE, KunShan Ultrasonic Instruments Co., Ltd., Jiangsu, China) for 10 min at 100 W and 20 kHz to obtain the desired PS concentrations (5, 10, 15, 20, 25, 30, 40, and 50 mg/L). The ZIF-67 used in this investigation was prepared through a high-throughput production method [47]. The concentrated hydrochloric acid (HCl) and sodium hydroxide (NaOH) used in this experiment were of analytical grade and were supplied by Tianjin Hengxing chemical reagent Co., Ltd., Tianjin, China.

### 2.2. Material Characterization

A Zetasizer Nano ZS system (Malvern Panalytical Co., Ltd., Malvern, UK) was used to measure the mean hydrodynamic diameters and Zeta potentials of ZIF-67 and PSMP particles at a 3–12 pH range. Fourier transform infrared spectroscopy (FTIR, tensor 27, Brooke, Germany) was applied to characterize the possible functional groups in PSMP and ZIF-67 (before and after plastic adsorption) at a 500–4000 cm^−1^ range. The surface morphologies of PSMPs and ZIF-67 (before and after adsorption) were observed via scanning electron microscopy (SEM, Zeiss GmbH, Oberkochen, Germany). Fluorescence images of PSMPs and ZIF-67 (before and after adsorption of plastics) were observed using a fluorescence microscope (NE620-FL, Ningbo Yongxin Optical Co., Ltd., Zhejiang, China).

### 2.3. Adsorption of PSMPs by ZIF-67

In the adsorption process, the ZIF-67 (with a constant mass) and PSMPs solutions (with different concentrations, 30 mL) were added into a conical glass flask (100 mL), then placed in an air bath oscillator (ZYC-1189L, Shanghai Zhetu scientific instrument Co., Ltd., Shanghai, China) at room temperature (25 °C) with a shaking speed of 150 rpm. Upon the completion of the shaking process, the concentration of PSMPs in the solution supernatant was determined with a fluorescence spectrometer (NF-900, Thermo Fisher Scientific, Seoul, Korea), with a scanning speed of 600 nm/min; the voltage of the photomultiplier tube was 400 V. The emission spectra were recorded at a 400–500 nm wavelength range after excitation at 468 nm.

All experiments were conducted in triplicate using independent samples. Conical glass flasks with a PSMP-only solution (without ZIF-67 addition) were set as the control group.

#### 2.3.1. Effect of ZIF-67 Dose and PSMP Concentration

To investigate the effect of the ZIF-67 dose on PSMP adsorption, various amounts of ZIF-67 (0.1, 0.2, 0.3, 0.4, 0.5, and 0.6 g/L) were added into the PS solution (5 mg/L, 30 mL) in a 150 mL Erlenmeyer flask. The contact time and temperature were 20 min and 298 K, respectively.

The PSMP adsorption rates of ZIF-67 (0.4 g/L) were then measured in response to various PSMP concentrations (5, 10, 15, 20, 25, 30, 40, and 50 mg/L). The contact time and temperature were 20 min and 298 K, respectively.

#### 2.3.2. Effect of Contact Time

The effect of reaction time on PSMP adsorption performance was assessed at different contact times (1, 5, 10, 20, 30, 40, 50, and 60 min) at a constant 0.4 g/L ZIF-67 dose. The volume of the PSMP solution (5 mg/L) was 30 mL, with a contact temperature of 298 K.

#### 2.3.3. Effect of pH and Temperature

The effect of pH on PSMP adsorption performance by ZIF-67 was investigated at a 3–12 pH range, whereas the other conditions remained constant (ZIF-67 dose of 0.4 g/L, contact time of 20 min, and 298 K temperature). The pH of the PSMP solution was adjusted with HCl (1 mol/L) or NaOH (1 mol/L). The effect of temperature on the PSMP (5 mg/L) adsorption by ZIF-67 (0.4 g/L) from the water environment was analyzed by setting the temperature at 288, 298, and 308 K, with a contact time of 20 min.

The PSMP adsorption rate (*η*) and capacity ($q_{t}$) of ZIF-67 were calculated using Equations (1) and (2), respectively:

(1) $$\eta =\frac{C_{0}-C_{t}}{C_{0}}\times 100\%$$

(2) $$q_{t}=\left(C_{0}-C_{t}\right)\times \frac{v}{m}$$

where *C*_0_ (mg/L) is the initial concentration of PSMPs; *C_t_* (mg/L) is the concentration of PSMPs in the solution after time *t* (min); *v* (L) is the volume of the PSMP solution; *m* (g) is the ZIF-67 dose in the solution.

### 2.4. Adsorption Model

The kinetics and isotherm models for PSMPs adsorbed by ZIF-67 were studied to explore the adsorption behavior of PSMPs particles by ZIF-67. Here, the pseudo-first-order model (Equation (3)) and pseudo-second-order model (Equation (4)) were used to fit the kinetic experimental results for PSMPs adsorbed by ZIF-67. Furthermore, the adsorption isotherms were fitted by the Langmuir model (Equation (5)) and Freundlich model (Equation (6)).

(3) $$q_{t}=q_{e}\left(1-e^{-k_{1}t}\right)$$

(4) $$q_{t}=\frac{q_{e}^{2}k_{2}t}{1+q_{e}k_{2}t}$$

(5) $$\frac{C_{e}}{q_{e}}=\frac{1}{k_{l}q_{m}}+\frac{1}{q_{m}}$$

(6) $$lnq_{e}=lnk_{F}+\frac{1}{n}lnC_{e\;\;\;}$$

where *q_e_* (mg/g) and *q_m_* represent the equilibrium and maximum theoretical adsorption capacity of PSMPs by ZIF-67, respectively; *k*_1_ (min^−1^) and *k*_2_ mg/(g × min) are the equation constants for the pseudo-first-order model and pseudo-second-order model, respectively. *K_l_* (L/mg) and *k_f_* are constants for the Langmuir model and Freundlich model, respectively; *n* is the Freundlich model constant; *q_m_* (mg/g) is the maximum theoretical adsorption capacity.

## 3. Results and Discussion

### 3.1. Material Characterization

**Figure 1:**
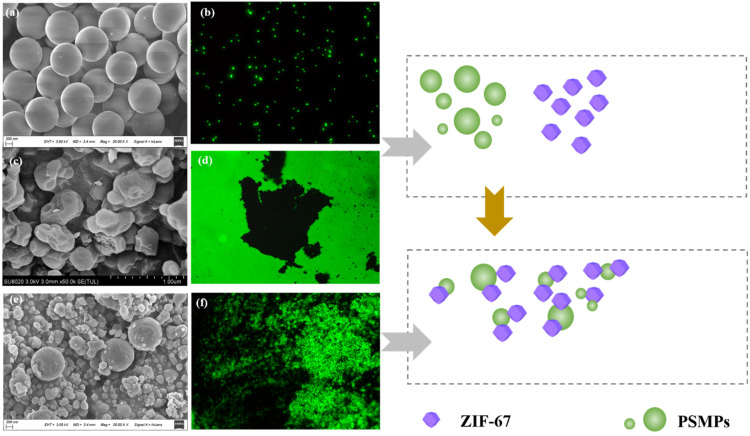
SEM and fluorescence image of PSMPs (**a**,**b**), ZIF-67 (**c**,**d**), and the ZIF-67/PSMP composite after adsorption (**e**,**f**), and schematic representation of PSMP adsorption by ZIF-67.

The SEM and fluorescence images of the PSMPs, ZIF-67, and the ZIF-67/PSMP composite after adsorption are illustrated in Figure 1. As shown in the figure, the original PSMPs exhibited a smooth surface and uniform spherical shape, with particle sizes ranging from 1.0 to 3.0 μm. As demonstrated by both the SEM and fluorescence images, the ZIF-67 particles were irregular, with diameters ranging from 30 to 600 nm and a relatively smooth surface. However, compared with the PSMPs, the fluorescence of ZIF-67 was markedly weaker and was almost negligible. After adsorption, the fluorescence of the ZIF-67/PSMP composite in the aqueous solution was markedly increased, indicating that the PSMPs had attached to the surface of ZIF-67. These findings further confirmed that the PSMPs particles were effectively adsorbed by ZIF-67 in the aqueous solution.

**Figure 2:**
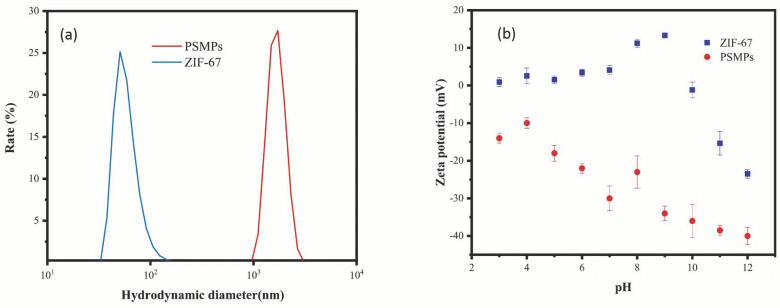
Hydrodynamic diameter distribution (**a**) and Zeta potential (**b**) of PSMPs and ZIF-67 in aqueous solution.

Figure 2a illustrates the hydrodynamic diameter distribution for the single ZIF-67 and PSMPs. As shown in the figure, the average diameters of ZIF-67 and PSMPs were approximately 92 nm and 1.45 μm, respectively. The Zeta potential (Figure 2b) of ZIF-67 and PSMPs were significantly different at a pH range of 3–12. The Zeta potential of the PSMPs was almost negative at the aforementioned pH range due to the presence of sulfate in the PSMPs [48,49]. Sulfate groups are negatively charged at a 2–12 pH range [48,49,50]. However, at a 6–10 pH range, the Zeta potential of ZIF-67 increased, and ZIF-67 became positively charged. Once the pH exceeded 10, ZIF-67 tended to be negatively charged, which was attributed to the OH^−^ derived from water and hydrolysis of uncoordinated Co^2+^ on the surface of ZIF-67 [47,51]. These variations in the Zeta potential of ZIF-67 and PSMPs in aqueous solution might determine the PSMP removal performance of ZIF-67.

**Figure 3:**
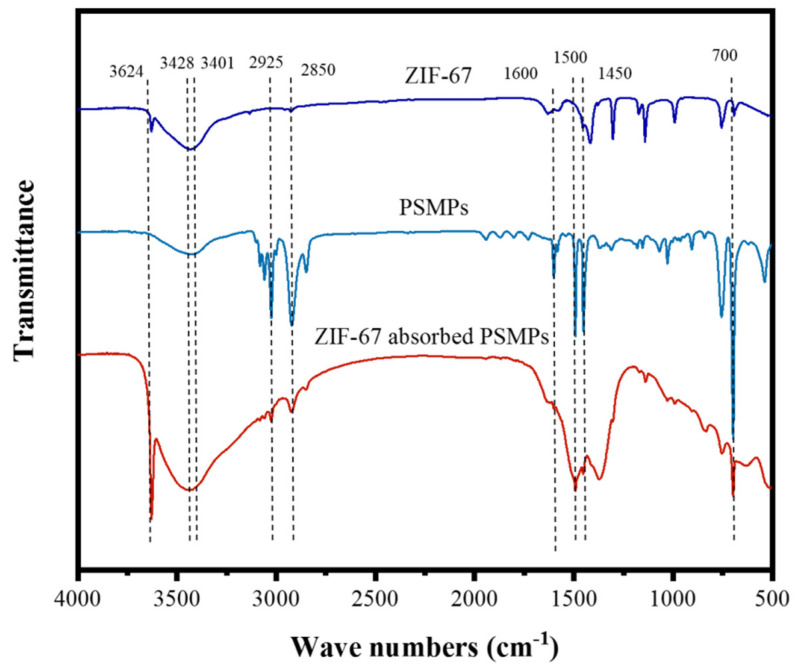
FTIR spectra for PSMPs and ZIF-67 during the adsorption process.

The FTIR spectra for PSMPs and ZIF-67 during the adsorption process are illustrated in Figure 3. For PSMPs, the absorption peak at 700 cm^−1^ was attributed to the single substitution peak of the benzene ring [52]. The peaks for PSMPs at 1450, 1500, and 1600 cm^−1^ were attributed to the skeleton vibration of the benzene ring [53,54]. Moreover, the peaks at 2850, 2925, and 3031 cm^−1^ in the PSMPs were mainly attributed to the C-H stretching of aliphatic compounds, whereas the peak at 3420 cm^−1^ was assigned to -OH in the PSMPs [55,56]. The FTIR peak for ZIF-67 was mainly ascribed to the ligand 2-methyl in the MOF material [57]. The peak observed at 600–1500 cm^−1^ was attributed to the stretching vibration of the imidazole ring in ZIF-67, whereas the peak at 3428 cm^−1^ was linked to the stretching vibration of N-H and -OH [43]. After the adsorption process, the characteristic peaks for PSMPs (700, 1450, 1500, 1600, 2850, 2925, 3031, and 3420 cm^−1^) were observed in the ZIF-67/PSMP composite, thus confirming that the PSMPs were adsorbed on the surface of ZIF-67. Additionally, the peak at 3300–3700 cm^−1^ in the ZIF-67 (after PSMP adsorption) was mainly related to the vibration of hydroxyl groups [51]. In turn, this hydroxyl vibration was mainly ascribed to the interaction between Co-OH or -NH (in ZIF-67) and the -OH (in PSMPs) [43,47,58]. Furthermore, the imidazole ring (in ZIF-67) could be regarded as an aromatic compound, which could interact with other aromatic compounds (in PSMPs) through π-π stacking interaction [43]. Therefore, the π-π stacking interactions should also be presented between the ZIF-67 and in PSMPs in an aqueous solution [59,60]. Therefore, hydrogen bonding and π-π stacking strengthened the interaction between ZIF-67 and PSMPs in the aqueous solution.

### 3.2. Effect of ZIF-67 Dose

**Figure 4:**
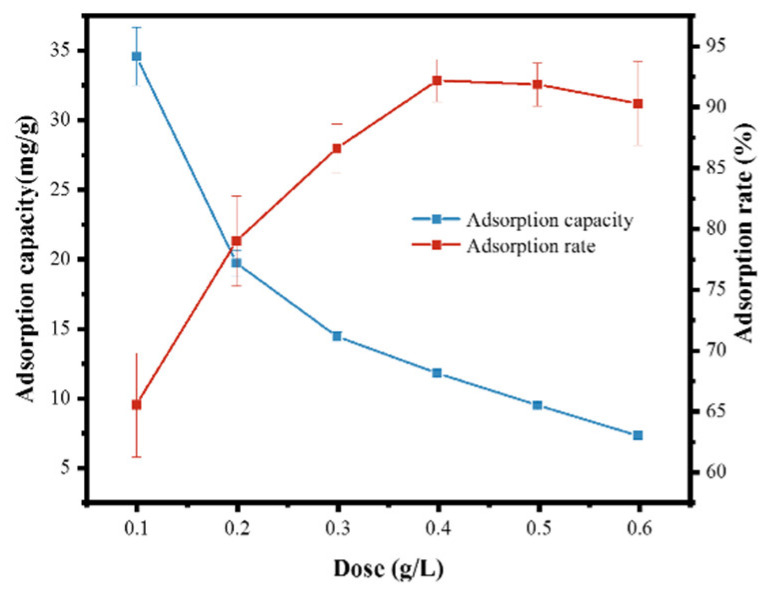
Effect of ZIF-67 dose on adsorption ratio and capacity of PSMPs in an aqueous solution. Conditions: PSMP concentration, 5 mg L^−1^; temperature, 298 K; reaction time, 20 min.

Figure 4 illustrates the effect of the ZIF-67 dose on PSMP removal in aqueous solution. At a ZIF-67 dose ranging from 0.1 to 0.6 g/L, the adsorption ratio of PSMPs by ZIF-67 increased from 65.4% to 90.2%. It could be attributed to the fact that more ZIF-67 could provide more adsorption sites for PSMPs. However, once the ZIF-67 dose exceeded 0.4 g/L, the adsorption ratio remained largely constant. Furthermore, the adsorption capacity of PSMPs by ZIF-67 decreased from 34.5 to 7.2 mg/g when the ZIF-67 dose increased from 0.1 to 0.6 g/L (Equation (2)). The highest adsorption rate (92.1%) and adsorption capacity (11.6 mg/g) of PSMPs in an aqueous solution was observed at a ZIF-67 dosage of 0.4 g/L. Thus, the optimal dosage of ZIF-67 for the removal of PSMPs should be selected as 0.4 g/L.

### 3.3. Adsorption Kinetics Model of PSMPs by ZIF-67

**Figure 5:**
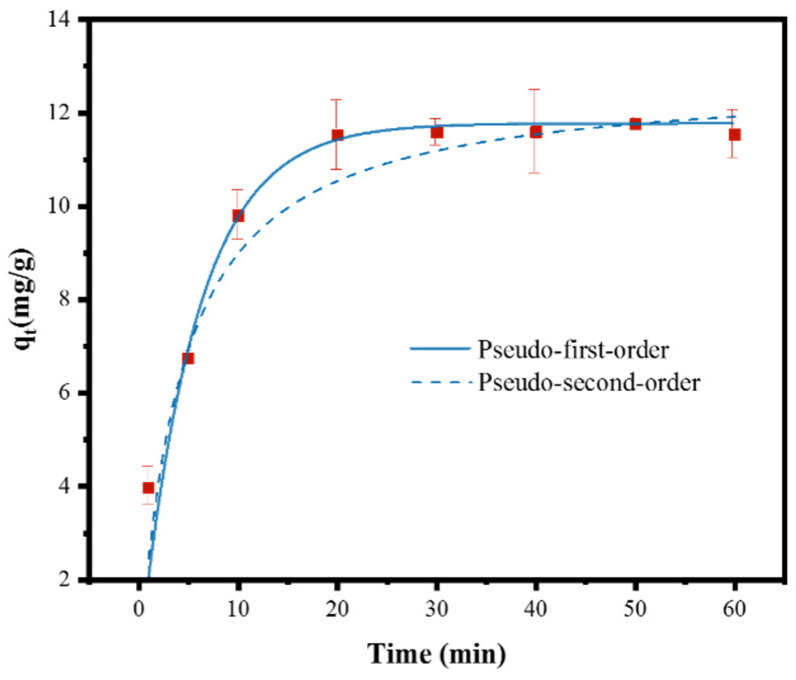
Fitting kinetic model for PSMP adsorption by ZIF-67. Conditions: PSMP concentration, 5 mg L^−1^; ZIF-67 dose, 0.4 g/L; temperature, 298 K, reaction time, 20 min.

Figure 5 illustrates the adsorption capacity of PSMPs by ZIF-67 with increasing contact time. In the first 20 min, the PSMPs would be rapidly adsorbed by ZIF-67, reaching a maximum adsorption capacity of 11.6 mg/g. Once the contact time exceeded 20 min, the PSMP adsorption capacity of ZIF-67 remained largely constant. Our findings thus indicated that the adsorption of PSMPs by ZIF-67 in aqueous solution is a relatively fast process.

The experimental results for the adsorption of PSMP by ZIF-67 were fitted by the pseudo-first-order and pseudo-second-order models, as shown in Figure 5 and Table 1. The calculated R^2^ for the pseudo-second-order kinetic model (0.981) was close to that of the pseudo-first-order model (0.977). However, the theoretical equilibrium adsorption capacity (11.7 mg/g) calculated by the pseudo-first-order kinetic equation was closer to the actual adsorption capacity (11.6 mg/g). Therefore, the pseudo-first-order kinetic equation was deemed more suitable for describing the adsorption of PSMPs by ZIF-67. These findings indicate that the removal of PSMPs by ZIF-67 is largely mediated by physical adsorption [61]. The adsorption rate constant of ZIF-67 for PSMPs was 0.171 min^−1^, which meant that ZIF-67 could reach the adsorption equilibrium for PSMPs within a short time [62].

### 3.4. Effect of pH

**Figure 6:**
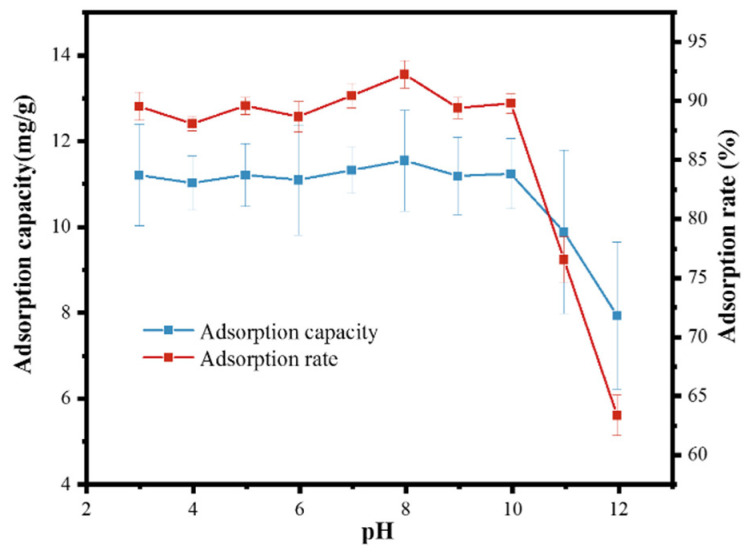
Effect of pH on PSMP adsorption capacity and rate by ZIF-67 (conditions: PSMP concentration, 5 mg L^−1^; ZIF-67 dose, 0.4 g/L; temperature, 298 K; reaction time, 20 min).

**Figure 7:**
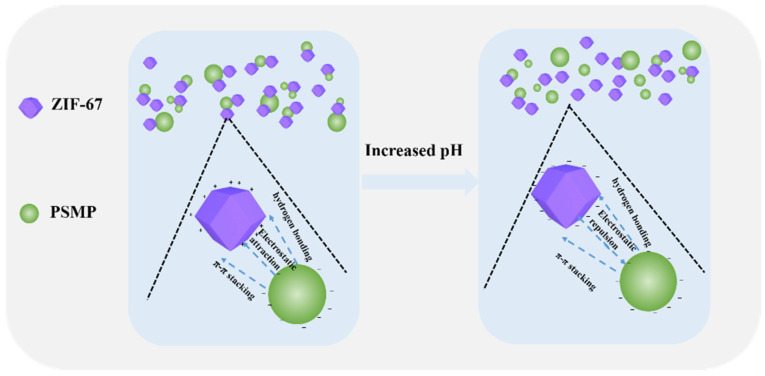
The schematic of the adsorption of PSMPs by ZIF-67 with the influence of increasing pH.

Figure 6 illustrates the effect of pH on the adsorption of PSMPs by ZIF-67 in an aqueous solution. The schematic of the adsorption of PSMPs by ZIF-67 with the influence of increasing pH was shown in Figure 7. As shown in the figure, the adsorption rate of PSMPs by ZIF-67 was maintained at approximately 88.3% at a 3–10 pH range. This high PSMPs adsorption rate at the aforementioned pH range was mainly attributed to the stronger electrostatic attraction between the positively charged ZIF-67 and the negatively charged PSMPs. Moreover, the π-π stacking and hydrogen bonding between the ZIF-67 and PSMPs also likely improved the removal of PSMPs in aqueous solution. When pH increased further to an 11–12 range, the repulsion force between the negatively charged ZIF-67 and negatively charged PSMPs at strongly alkaline conditions reduced the removal ratio of PSMPs by ZIF-67 from 88.3% to 64.4%. Therefore, ZIF-67 could effectively adsorb PSMPs with a pH ranging from 3 to 10, while the highest PSMPs adsorption rate was observed at a pH around 8.

### 3.5. Adsorption Isotherm Analysis

**Figure 8:**
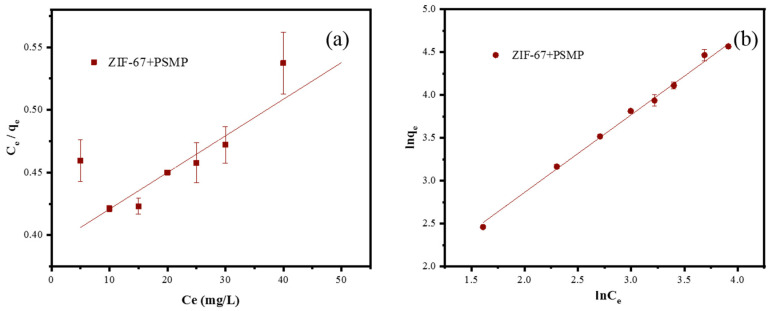
Fitting adsorption isotherm models of PSMPs by ZIF-67 (conditions: PSMPs concentration, 5 mg L^−1^; ZIF-67 dose, 0.4 g/L; temperature, 298 K; reaction time, 20 min). (**a**) Langmuir model; (**b**) Freundlich model.

The adsorption isotherm for the adsorption of PSMPs by ZIF-67 in an aqueous solution was also investigated (Figure 8); the fitting parameters are summarized in Table 2. The fitting coefficient (R^2^) for the Freundlich model (0.997) was much greater than that of the Langmuir model (0.474), indicating that the Freundlich model was more suitable to describe the adsorption of PSMPs by ZIF-67 in the aqueous solution. Additionally, this confirmed that the adsorption process is controlled by a multilayer adsorption mechanism. The fitting model results also indicated that the surface adsorption energy of ZIF-67 was uneven and that the adsorption of PSMPs by ZIF-67 could be attributed to hydrophobicity [36,63,64]. In the Freundlich model, the *k_f_* value and the 1/n value indicated the favorability of ZIF-67, to interact with PSMPs [65]. Thus, the value of 1/n (0.327) and *k_f_* (2.58) confirmed that ZIF-67 would readily interact with PSMPs [64,65]. The ZIF-67 would maintain a high adsorption rate (>82.2%) with ranging PSMPs concentrations (5 mg/L-50 mg/L), indicating a superior state for removal of PSMPs by the ZIF-67.

### 3.6. Effect of Temperature

**Figure 9:**
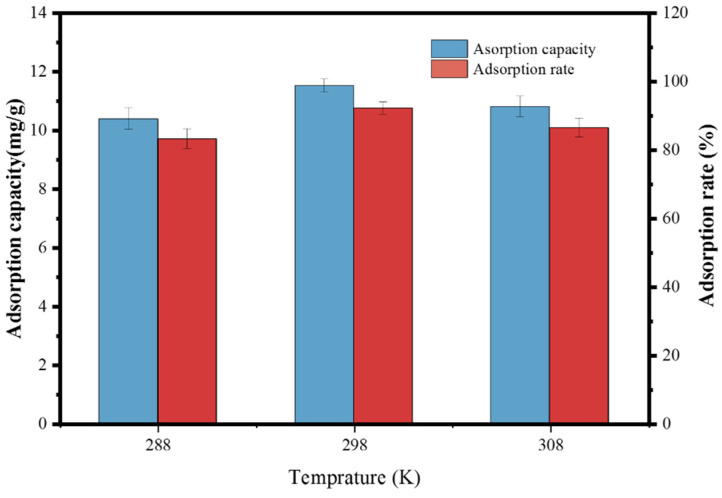
Effect of temperature on the PSMP adsorption performance of ZIF-67 (conditions: PSMP concentration, 5 mg L^−1^; ZIF-67 dose, 0.4 g/L; contact time, 20 min).

Figure 9 illustrates the effect of contact temperature on the adsorption of PSMPs by ZIF-67. When the contact temperature ranged from 288 to 308 K, the PSMP adsorption rate by ZIF-67 in the aqueous solution first increased slightly (from 83.1% to 92.1%) and then decreased (from 92.1% to 86.4%). This increase in adsorption capacity (from 10.3 mg/g to 11.5 mg/g) with increasing contact temperature (from 288 K to 298 K) was linked to the fact that the adsorption of PSMPs by ZIF-67 was mainly driven by physical adsorption (particularly at 288 K). Here, the adsorption of PSMPs by ZIF-67 was mainly attributed to chemical adsorption, whereas the number of adsorption active sites at 298 K increased with increasing contact temperature. However, the slight decrease in adsorption capacity observed in our experiments (from 11.5 to 10.6 mg/g) was likely due to the desorption of PSMPs from the ZIF-67 due to the increase in the thermal motion of particles with increasing temperature [66,67]. Therefore, our findings confirmed that increasing the temperature negatively affects the removal of MPs by MOF materials in aqueous solution. Furthermore, the relatively higher adsorption rate (92.1%) of PSMPs by ZIF-67 was determined at a contact temperature of 298 K. The correlation observed between adsorption rate and temperature was not obvious; thus, the Freundlich model constants at varying temperatures could not be calculated. Therefore, the thermodynamic results would not be further discussed.

### 3.7. Compared with Other Treatments Technologies

Different treatment technologies for removing microplastics are summarized in Table 3, including the adsorption, flocculation, electrocoagulation, and MBR route. For the flocculation method, the additional chemical reagents of PAC (200 mg/L) and PAM (100 mg/L) would be added, with MPs removal rate of 54.7% during the flocculation process [68]. Higher operating cost would be consumed in the MBR system, with PE/PET removal efficiency of 98.5% within 35 h [69]. The electrocoagulation route would remove more than 91% of microplastics with an applied voltage density of 10 V [70]. In addition, the electrocoagulation process would require Na_2_SO_4_ (0.05 M) to improve the conductivity of the solution [70,71]. The activated sludge process or biodegradation route had a relatively lower operating cost and wider application range; however, they took a longer time (more than 56 days) and the removal rate of microplastics still needed to be improved [72,73]. M-CNTs (5 g/L) could nearly remove all of PSMPs (5 g/L) in an aqueous solution, and the reaction time was up to 5 h. From the above comparison, the ZIF-67 materials have advantages including a short reaction time (20 min), relatively higher removal efficiency (92.1%), and a simple operation process.

## 4. Conclusions

This paper mainly focused on the mechanism for interface between microplastics and the prepared MOF materials. The current experiment mainly studied the influence mechanism of single key factors on the adsorption of microplastics. Our research showed that the ZIF-67 could effectively absorb different concentrations of PSMPs (5–50 mg/L) in a wide pH range (3–10), and a temperature ranging from 288 to 308 K. In addition, compared with other technologies for removing PSMPs from water, ZIF-67 showed the superiority including a lower cost and higher treatment efficiency.

This process was a challenging attempt, which not only provided a novel technology for effectively removing microplastics in acidic and weakly alkaline wastewater, but also expanded the application of porous materials in removing pollutants in aqueous solutions. Further tests would be recommendable to verify the reusability of MOF materials in the adsorption process. We would also add more experimental groups to the further investigation.

## Figures and Tables

**Table 1:** Kinetic model and parameters of adsorption of PSMPs by ZIF-67.

Adsorbent	Pseudo-First-Order			Pseudo-Second-Order		
	$q_{e}$	$K_{1}$	R^2^	$q_{e}$	$K_{2}$	R^2^
ZIF-67	11.7	0.171	0.977	12.7	0.018	0.981

**Table 2:** Adsorption isotherm models and parameters of adsorption of PSMPs by ZIF-67.

Adsorbent	Langmuir			Freundlich		
	$K_{1}$	$q_{m}$	R^2^	$K_{f}$	1/n	R^2^
ZIF-67	0.002	1369.8	0.474	2.58	0.346	0.997

**Table 3:** Methods for the removal of microplastics.

Order	Type of Microplastics	Method	MP Size	Removal Efficiency	Experimental Details	Reference
1	PS	Adsorption by ZIF-67	1.45 μm	92.1%	MPs (5 mg/L), ZIF-67(0.4 g/L), contact temperature (298 K), contact time (20 min)	This work
2	PE, PET, PA	Adsorption by M−CNTs	48 μm	100%	MPs (5 g/L), M−CNTs (5 g/L), contact temperature (298 K), contact time (5 h)	[74]
3	PET	Coagulation	100–400 μm	54.7%	PAC (100 mg/L), PAM (200 mg/L), MPs (100 mg), stirring speed of 500 rpm for 1 min and followed by 100 rpm for 15 min	[68]
4	PA, PP, PE, PVC	Activated sludge	0.02–0.3 mm	64.4%	The abundance of MPs in the influent (79.9 n/L)	[72]
5	PS	Biodegradation	0.3–1.25 mm	43.7%	Basal medium (liquid carbon-free basal medium), temperature (40 °C or 70 °C), contact time (56 days)	[73]
6	PMMA, PE, CA, PP	Electrocoagulation	PE: 286.7 μm, PMMA: 6.3 μm, PP: 1–2 mm, CA:1–2 mm	93.2% for PE, 91.7% for PMMA, 98.2% for CA, and 98.4% for PP	Electrolyte (0.05 M Na_2_SO_4_), pH (7.2), applied voltage density (10 V), anode (Al)	[70]
7	PE, PET	MBR	<5 mm	98.5%	MBR pilot plant, Suction cycle(9 min-ON/1 min-OFF), HRT(35 h)	[69]

## Data Availability

Not applicable.

## Abbreviations

PS	Polystyrene
MPs	Microplastics
PSMPs	Polystyrene microplastics with micron size
MOF	Metal–organic framework
ZIF	Zeolitic imidazolate framework
XRD	X-ray diffraction
FTIR	Fourier transform infrared
SEM	Scanning electron microscope
PE	Polyethylene
PET	Polyethylene terephthalate
PA	Polyamide
M-CNTs	Magnetic carbon nanotubes
PAM	Polyacrylamide
PAC	Poly-aluminum chloride
PP	Polypropylene
PVC	Polyvinyl chloride
PMMA	Polymethylmethacrylate
CA	Cellulose acetate
MBR	Membrane bioreactor
HRT	Hydraulic retention time
